# Utilizing AI for decision support in severe asthma biological treatment

**DOI:** 10.3389/fdgth.2026.1939100

**Published:** 2026-10-30

**Authors:** Bassam Mahboub, Sameer Alawnah, Salam Dhou, Piotr Kuna, Celine Bergeron, Riyad Al-Lehebi, Najla Alnahadi, Noura Alhousani, Assim Sagahyroon, Asharaf Alzabi, Laila Salameh

**Affiliations:** 1 Pulmonary Medicine, Dubai Health, Dubai, United Arab Emirates; 2 Department of Computer Science and Engineering, American University of Sharjah, Sharjah, United Arab Emirates; 3 Division of Internal Medicine Asthma and Allergy, Medical University of Lodz, Lodz, Poland; 4 Department of Medicine, Centre for Lung Health, Vancouver General Hospital, Vancouver, Canada; 5 Department of Medicine, University of British Columbia, Vancouver, Canada; 6 Department of Pulmonology, King Fahad Medical City, Riyadh, Saudi Arabia; 7 College of Medicine, Alfaisal University, Riyadh, Saudi Arabia; 8 Respiratory Medicine Division, Zayed Military Hospital, Abu Dhabi, United Arab Emirates; 9 Department of Internal Medicine, College of Medicine and Health Sciences, United Arab University, Alain, United Arab Emirates; 10 Pulmonary Department, Mediclinic, Abu Dhabi, United Arab Emirates; 11 Research & Graduate Studies Department, Mohammed Bin Rashid University of Medicine and Health Sciences, Dubai Health, Dubai, United Arab Emirates

**Keywords:** arteficial intelligence, asthma, biological theraphy, precision medicine, severe asthma

## Abstract

**Background:**

Severe asthma remains a major clinical challenge because of disease heterogeneity and variable response to biologic therapies. Despite the availability of clinical guidelines, clinicians often rely on a trial-and-error approach when selecting biologic treatments. We aimed to develop and validate an artificial intelligence (AI)-driven decision support system (DSS) to predict treatment response and support personalized biologic selection in severe asthma.

**Methods:**

A retrospective multinational cohort of 801 patients with severe asthma from the United Arab Emirates, Saudi Arabia, Poland, and Canada was analyzed. Random Forest (RF) models were developed to predict clinical improvement, and Recursive Feature Elimination was used to identify the most informative predictors. Counterfactual simulation was subsequently applied to evaluate alternative biologic treatment strategies.

**Results:**

The most important predictors of treatment response were Asthma Control Test (ACT) score, eosinophil count, and type 2 inflammatory comorbidities, particularly nasal polyposis and sinusitis. The optimized RF model improved specificity from 33.33% to 66.67% and increased overall accuracy from 78.57% to 82.14%. Counterfactual simulations demonstrated the model's ability to identify non-responder phenotypes and estimate alternative biologic options with higher predicted probabilities of improvement, including a 62% probability of improvement following a switch from anti-IgE therapy to anti-IL-5R therapy in selected patients.

**Conclusion:**

This study demonstrates the potential of AI-driven clinical decision support to support personalized biologic selection in severe asthma. By improving identification of likely non-responders and simulating alternative treatment pathways, the proposed framework may help reduce ineffective treatment exposure, improve clinical decision-making, and advance precision medicine in severe asthma management.

## 1 Introduction

Asthma is a heterogeneous disease characterized by a range of clinical symptoms as well as bronchial hyperresponsiveness or reversible expiratory airflow restriction. It is estimated that the condition affects around 24 million people in the United States and approximately 300 million people worldwide, and its frequency has been rising (1, 2). Up to 50% of patients are acknowledged to be poorly controlled despite guideline-based therapy, and 5% to 10% of patients have a particularly severe illness that is frequently resistant to standard care (3, 4).

Severe asthma is a major burden on individuals and healthcare systems since it is characterized by frequent exacerbations and prolonged symptoms even after high-dose inhaled medication (5). Due to their severe asthma, they are now being considered as patients for biological treatment (6). Biological medicines that target inflammatory pathways have transformed the treatment of severe asthma by improving symptom control and lowering exacerbations. Several pathways have gained interest and resulted in the creation of new targeted interventions. These consist of: (a) IgE (omalizumab), (b) eosinophils signaling pathways; IL-5 (Mepolizumab) and IL-5R (Benralizumab), (c) TSLP signaling pathway (Tezepelumab), and (d) the IL-4/IL-13 signaling pathway (Dupilumab) (7).

Although these biologics have revolutionized treatment, a significant issue persists in clinical practice: the “endotype gap.” Although many biologics focus on “T2-high” asthma (marked by elevated eosinophils and IgE), patients frequently exhibit overlapping characteristics or “T2-low” (non-eosinophilic) profiles. Existing clinical guidelines offer a structure for decision-making, yet healthcare providers lack accurate tools to evaluate these biological markers in relation to multi-national, real-world data like regional comorbidities, genetic vulnerabilities, and varied demographic profiles (8).

However, because responses to biologic therapies vary greatly depending on a number of patient factors, such as underlying asthma phenotype, comorbidities, and genetic predispositions, choosing the best biologic for a given patient is complicated and still a clinical challenge. Because of this intricacy, physicians occasionally choose biologics by trial and error, which can postpone the best course of treatment and worsen patient outcomes. The use of cutting-edge technologies, especially artificial intelligence (AI). AI denotes the capacity of a machine or system to carry out activities that necessitate human intelligence, including reasoning, learning, and making decisions (9). AI includes a range of technologies, including machine learning, deep learning, natural language processing, robotics, speech recognition, and other automation tools (10). In the field of clinical decision making, AI has shown impressive abilities to forecast and categorize diagnoses, in addition to offering recommendations and insights (10, 11). Growing empirical evidence indicates that knowledge-driven computerized decision support (CDS) and specifically knowledge-driven clinical decision support systems can significantly improve practitioner performance (12, 13). Machine learning (ML) models can identify patterns in large datasets and predict outcomes with high accuracy (14).

This will help clinicians make better decisions and has grown in popularity as a solution to this problem. By evaluating enormous volumes of data to find trends, forecast treatment outcomes, and optimize therapeutic choices, AI-based decision support systems have demonstrated a great deal of promise in enhancing healthcare outcomes (14).

In order to suggest the best biologic therapy for the treatment of severe asthma, AI models can examine patient data, such as demographic traits, clinical parameters, and previous treatment outcomes. Trial-and-error methods can be lessened, clinical decision-making can become more precise, and patient outcomes can be improved in the end. In this study, we aim to develop an AI-based recommender system to assist healthcare providers in selecting the most effective biological treatment for severe asthma patients. This decision support system (DSS) uses anonymized patient data, including demographics, clinical characteristics, and treatment history, to predict treatment efficacy and provide personalized recommendations.

This study aimed to create and validate a multi-country, AI-driven decision support system that utilizes real-world clinical data to accurately predict therapeutic non-responders and simulate tailored biologic switching options.

## 2 Materials and methods

In this section, the materials and methods used in this study are described. Section 2.1 describes the data sets used in this study, which Section 2.2 describes the methods used.

### 2.1 Datasets

A retrospective cohort study was conducted using anonymized data from 801 severe asthma patients from patients were of mixed nationalities UAE, Saudi Arabia, Poland, and Canada. The dataset comprised patient demographics (age, gender, BMI,), asthma-related information (current medication, exacerbation history, eosinophil count, FEV1), and coexisting conditions (e.g., cancer, thyroid disease, eczema). Treatment responses were quantified using several metrics, including the Asthma Control Test (ACT), the number of exacerbations, oral corticosteroid (OCS) usage, and spirometry results (FEV1). The primary outcome “Overall Improvement,” was characterized as a binary variable (Improved/Not Improved). In order to maintain clinical consistency and reduce physician bias, “Improved” was precisely defined as achieving at least two of the subsequent criteria: (1) a clinically meaningful rise in ACT score (≥3 points), (2) *a* ≥ 50% decrease in the yearly exacerbation rate, or (3) a notable decrease in OCS use yearly.

### 2.2 Methods

In this study, four primary tasks were undertaken:

Task 1 (Baseline): Baseline Predictive Modeling of Clinical Outcomes.

Task 2 (Selection): Feature Importance Analysis and Selection.

Task 3 (Optimization): Enhanced Predictive Modeling Using a Reduced Set of Selected Features identified in Task 2.

Task 4 (Counterfactual Simulation): Prediction of Optimal Biological Agent Using a Counterfactual Simulation Approach, these tasks are demonstrated in Figure 1 and described in detail in the following subsections.

**Figure 1:**
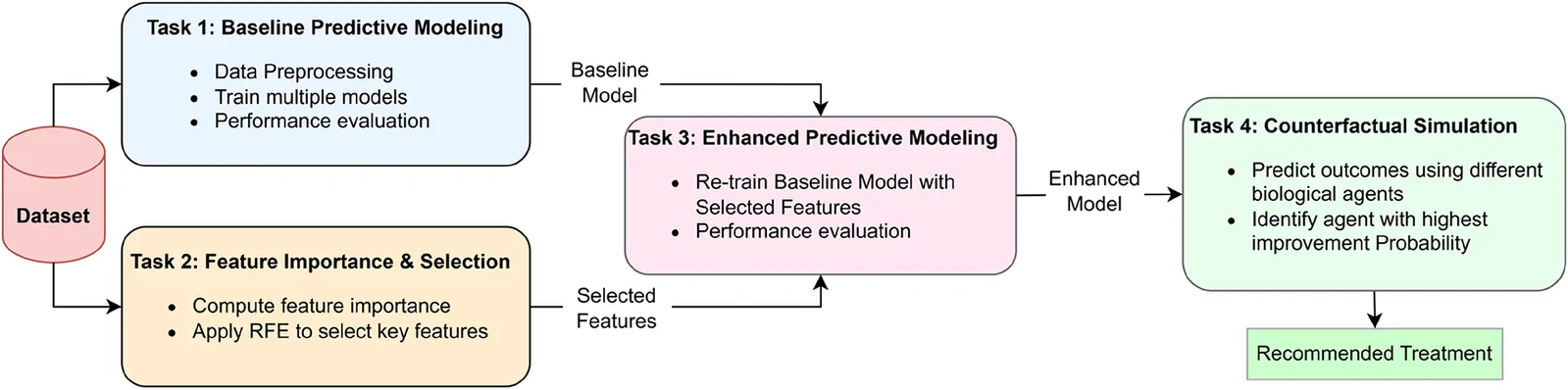
Overview of the proposed methodology.

#### 2.2.1 Task 1 (baseline): baseline predictive modeling of clinical outcomes

In this task, a binary classification model is built to predict the overall improvement status (Improved vs. Not Improved) based on the complete patient profile as described in Section 2.1. This “comprehensive-features” method sets a foundational performance level for the system, employing all accessible clinical and demographic data points (such as age, BMI, FEV1, eosinophil counts, and comorbidities) to assess if a patient is positively will respond to their assigned biological therapy.

Several steps were performed to accomplish this task. These steps include data preprocessing, training several classification models, testing and evaluating these models.

### 2.3 Data preprocessing

In the data preprocessing module, several operations were performed. First, the records with missing values were dropped from the dataset. As a result, only 7 records with missing values out of 801 (0.87%) were dropped. Feature encoding was applied to convert the categorical attributes to numerical ones so they can be as input in the machine learning models.

After these data preprocessing steps, the preprocessed dataset was divided into training and testing subsets, with five-fold cross-validation applied on the training dataset. In cross-validation, the dataset was divided into five folds (i.e., subsets), using one of these folds as a validation set, and training the model on the remaining folds at each run. Finally, the results resulting from each run are averaged to produce a robust estimate of the model's performance.

### 2.4 Training multiple classification models

The objective of this step is to identify the most suitable algorithm and the most appropriate performance metric (e.g., macro recall, micro recall, F1 score) to use as the optimization criterion. To achieve this, a repeated hold-out approach was employed. During the model development phase, a repeated hold-out strategy was employed in which the dataset was randomly split into 80% training and 20% testing subsets. This configuration was used After selecting the optimal model architecture and hyperparameters, a final independent evaluation was conducted using 93% training and 7% hold-out testing split. This larger training proportion was chosen to maximize data utilization for model learning while preserving a strictly unseen test set for final performance reporting.

At each run, cross-validation and extensive hyperparameter tuning were applied to the training set to determine the most effective models. Several optimization criteria were explored during hyperparameter tuning, and macro recall (defined as the average of sensitivity and specificity) was found to yield the most acceptable performance. To convert predicted probabilities into binary class labels (“Improved” vs. “Not Improved”), a classification threshold was selected on the training data based on the optimization criterion (macro recall) and then applied unchanged to the independent test set and counterfactual simulations. As this threshold was not fixed at 0.50, predicted labels may not always correspond to probabilities above or below 0.50.

During the initial model development phase, several tree-based classification algorithms, including Random Forest (RF), CatBoost, XGBoost, and Gradient Boosting Machine (GBM), were explored for preliminary comparative evaluation. These models were assessed to support algorithm selection prior to final model construction. Based on comparative performance and stability across validation runs, Random Forest demonstrated the most consistent results and was therefore selected for all subsequent analyses. The remaining models were not advanced to the final evaluation stage and are not discussed further in the Results section.

### 2.5 Model selection and performance evaluation

The dataset was divided into 93% for training and 7% for testing, ensuring that samples from different biological agents were represented in both subsets. The RF algorithm, combined with cross-validated hyperparameter tuning, was applied to the training data using macro recall as the optimization criterion. The final model, with optimal hyperparameters, was selected based on the highest macro recall achieved. This model was then evaluated on the independent testing subset, which was not seen during training, and the corresponding results were reported.

The performance of the classification models was evaluated using multiple evaluation metrics, including accuracy, sensitivity, specificity, area under the receiver operating characteristic (ROC) curve (AUC), and the confusion matrix.

#### 2.5.1 Task 2 (selection): feature importance analysis and selection

In this task, feature analysis and selection are performed to transform the model from a “black box” into an understandable and interpretable clinical tool. By exploring the feature importances, crucial clinical factors that most influence treatment results are pinpointed. This task is essential for determining which biological indicators (such as eosinophil levels or IgE concentrations) are the most robust predictors of success among various patient phenotypes.

Recursive Feature Elimination (RFE) is used as a feature selection technique to systematically remove the least important features from a dataset to improve model performance, reduce overfitting, and enhance interpretability. Since tree-based methods were found to perform the best on this dataset, and particularly, random forest, the importance of using Gini index was the feature selection criteria considered for RFE technique.

Gini Index is a metric commonly used in decision tree-based algorithms to evaluate the importance of features in classification tasks. Gini Index measures the impurity of a decision tree node, quantifying how mixed the classes are within that node. Formally, the Gini index for a node is defined as:

$$\mathrm{Gini}=1-\overset{C}{\underset{i=1}{\sum }}\;p_{i}^{2}$$

Where *p_i_* is the proportion of samples belonging to class *i* in the node, and *C* is the number of classes. A lower Gini index indicates a purer node, where most samples belong to a single class. In decision tree construction, features that maximize the reduction in Gini index when splitting a node are considered more important.

For feature selection, the Gini index can be aggregated across all splits in a tree or ensemble (e.g., Random Forest) to compute a feature importance score. Features contributing to larger reductions in Gini impurity are deemed more informative for predicting the target. Gini index is a heuristic measure of purity. Its main advantage is the ability to capture non-linear relationships and interactions between features and the target, making it especially useful in tree-based models and high-dimensional datasets. Gini index is used to rank features according to their importance ability in distinguishing between classes.

#### 2.5.2 Task 3 (optimization): enhanced predictive modeling using a reduced set of selected features identified in task 2

In this task, the features selected using the RFE technique, using the Gini index as the feature selection criterion, were used as inputs to the RF model. To ensure a fair comparison with the RF model developed in Task 1, this RF model with feature selection was trained and evaluated using the same training and testing sets as in Task 1. Cross-validated hyperparameter tuning was applied to the training data using macro recall as the optimization criterion. The final model was selected based on the highest macro recall achieved. This model was then evaluated on the same independent testing subset used in Task 1, and the corresponding results were reported.

Similar to the model performance evaluation conducted in Task 1, the model in this task is evaluated using multiple metrics, including accuracy, sensitivity, specificity, AUC, and the confusion matrix.

#### 2.5.3 Task 4 (counterfactual simulation): prediction of optimal biological agent using a counterfactual simulation approach

In this task, the proposed method recommends the most effective biological agent from several treatment options for a given patient profile. This is achieved by applying a counterfactual simulation approach, where the biological agent in each patient's profile is systematically replaced with each available treatment option in the dataset.

For each modified (counterfactual) instance, the trained model is used to predict the clinical outcome and the probability of clinical improvement. Then, the biological agent associated with the highest predicted probability of improvement is recommended as the optimal treatment for that patient. The best-performing model from previous tasks, namely, the RF model trained in the selected features, is used for this analysis.

This counterfactual approach facilitates personalized treatment recommendations and supports clinical decision-making by estimating the expected effectiveness of each biological agent for individual patients.

##### 2.5.3.1 Implementation details and reproducibility

All analyses were conducted using Python (version 3.10). The primary machine-learning implementation relied on the scikit-learn library (version 1.2), along with NumPy (version 1.24) and pandas (version 1.5) for data preprocessing and analysis. Model development and evaluation were performed on standardized datasets using fixed random seeds to ensure reproducibility.

Random Forest classifiers were trained with cross-validated hyperparameter tuning on the training data, using macro recall as the optimization criterion. Recursive Feature Elimination (RFE) employed Gini impurity-based feature importance from the Random Forest model. The full dataset comprised 801 patients, with 625 classified as Improved (78.0%) and 176 classified as Not Improved (22.0%), reflecting an imbalanced outcome distribution.

### 2.6 Ethical consideration

The Dubai Scientific Research Ethics Committee (DSREC) of the Dubai Health Authority (DHA), United Arab Emirates, approved this research ethically (Approval No. DSREC-11/2019_10). Additional data contributors in Saudi Arabia, Canada, and Poland secured independent ethical approval from their local authorities before sharing data. Data were accessed for research purposes from 13/10/2024 to 30/01/2026.All patient information was anonymized at the source and transmitted through secure methods in accordance with the data protection laws of each country.

### 2.7 The AI disclosure

No artificial intelligence tools were used in writing this manuscript.

## 3 Results

In this section, we present the results of the proposed methodology, organized by task. For each task, we describe the analysis performed, the key findings, and the corresponding performance metrics. Section 3.1 reports the baseline predictive modeling results (Task 1), Section 3.2 summarizes the feature importance analysis and selection results (Task 2), Section 3.3 presents the results of enhanced predictive modeling using the selected features (Task 3), and Section 3.4 demonstrates the counterfactual simulation approach for recommending the optimal biological agent (Task 4).

### 3.1 Baseline predictive modeling results (task 1)

By employing the RF model considering all input features, The resulting confusion matrix (Table 1) corresponds to an independent test set of 56 patients, including 12 classified as “Not Improved”, with 40 true positives (TP) and 4 true negatives (TN). The model's showed high sensitivity (90.91%) reflecting a robust ability to recognize patients who experienced clinical improvement. Whereas the specificity was significantly low (33.33%), suggesting a challenge in correctly identifying “Not Improved” instances.

**Table 1:** Confusion matrix of the random Forest Model considering all input features

True	Prediction: Improved	Prediction: Not Improved
Improved	40	4
Not Improved	8	4

This led to a high Positive Predictive Value (83.33%) while the Negative Predictive Value was lower (50.00%). Although the model attained an average recall, reflecting both sensitivity and specificity, stood at 62.12%, an overall accuracy of 78.57% and a commendable AUC of 0.83. as seen in (Figure 2) the disparity between sensitivity and specificity indicates that the model is presently leaning towards the predominant “Improved” class.

**Figure 2:**
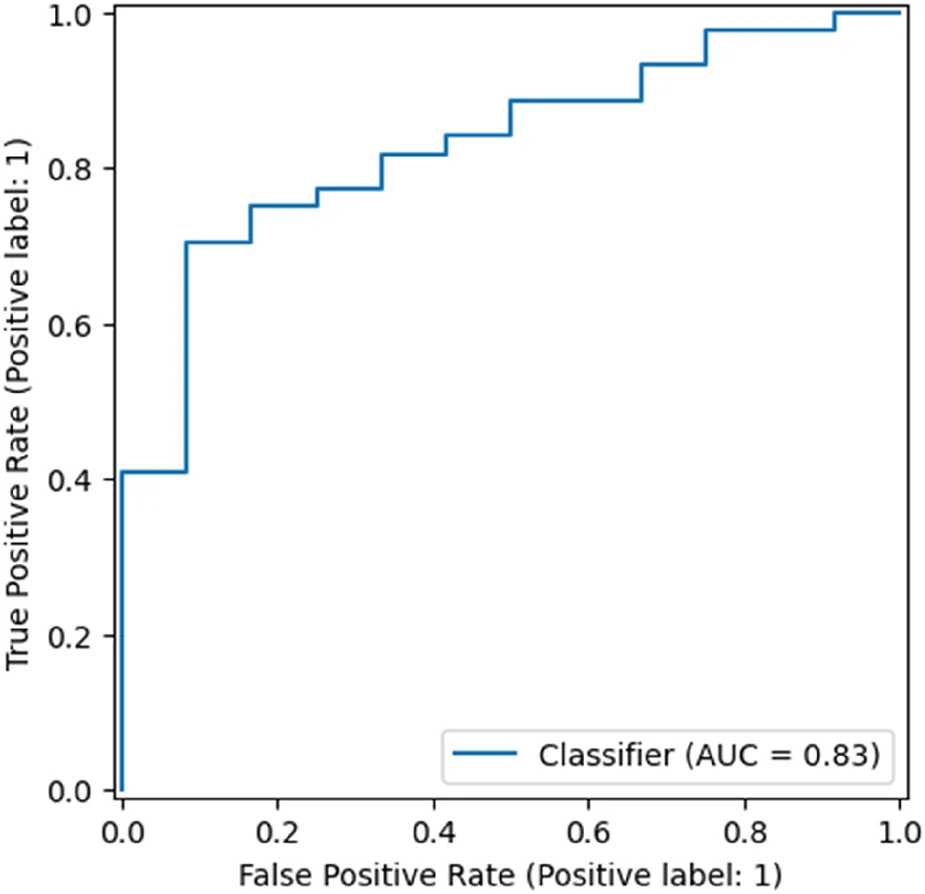
ROC curve and the achieved AUC result for RF based on all input features.

### 3.2 Feature importance analysis and selection results (task 2)

In this task, RFE was utilized to identify the key features that impact the model's prediction of treatment outcomes. The procedure methodically eliminated less important variables, retaining only those with the highest predictive power. The analysis resulted in 14 top-ranked features, as shown in Figure 3, with features removed by RFE indicated below the dashed line. The selected features include both variables linked to treatment and comorbid conditions that could greatly influence patient reactions to biologic therapy.

**Figure 3:**
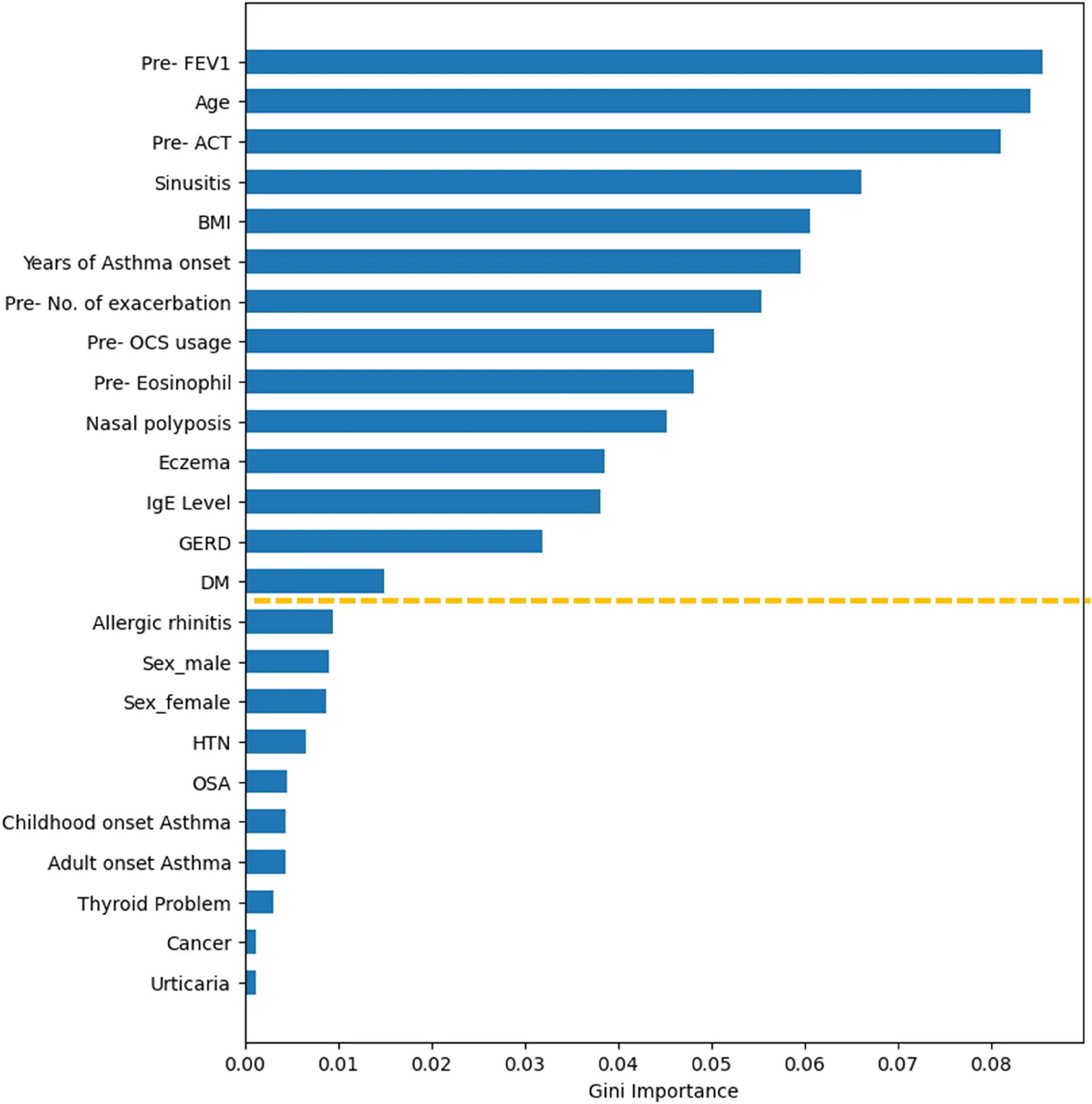
Ranked features and their scores according to gini importance criterion.

Out of the metrics quantifying treatment responses were found to be key predictors such as spirometry results (FEV1), Asthma Control Test (ACT), the number of exacerbations, oral corticosteroid (OCS) usage. Clinical factors such as eosinophil count, along with patient demographics, particularly age and BMI, were also found to be important predictors. Comorbid conditions, including sinusitis and eczema, were identified as significant predictors, whereas OSA, GERD, cancer, urticaria, and thyroid disorders were not. Gender was likewise not found to be a significant predictor.

### 3.3 Enhanced predictive modeling results (task 3)

Utilizing the most important features which were identified in Task 2, the RF model achieved improved performance with an overall accuracy of 82.14% (Table 2). The model showed a sensitivity of 86.36% and a notably enhanced specificity of 66.67%, effectively addressing the class-imbalance issues identified in the baseline model. However, given the limited number of “Not Improved” cases in the independent test set, specificity and negative predictive value estimates are sensitive to sampling variability and should be interpreted cautiously.

**Table 2:** Confusion matrix of the random Forest Model considering selected input features.

True	Prediction: Improved	Prediction: Not Improved
Improved	38	6
Not Improved	4	8

Although the Positive Predictive Value (PPV) was strong at 90.48%, the Negative Predictive Value (NPV) was relatively low at 57.14%. The average recall, reflecting both sensitivity and specificity, achieved 76.52%. Examination of the confusion matrix showed 38 True Positives and 8 True Negatives, demonstrating a more balanced discriminatory ability between responders and non-responders. The overall diagnostic performance of the model is also demonstrated by the AUC shown in Figure 4.

**Figure 4:**
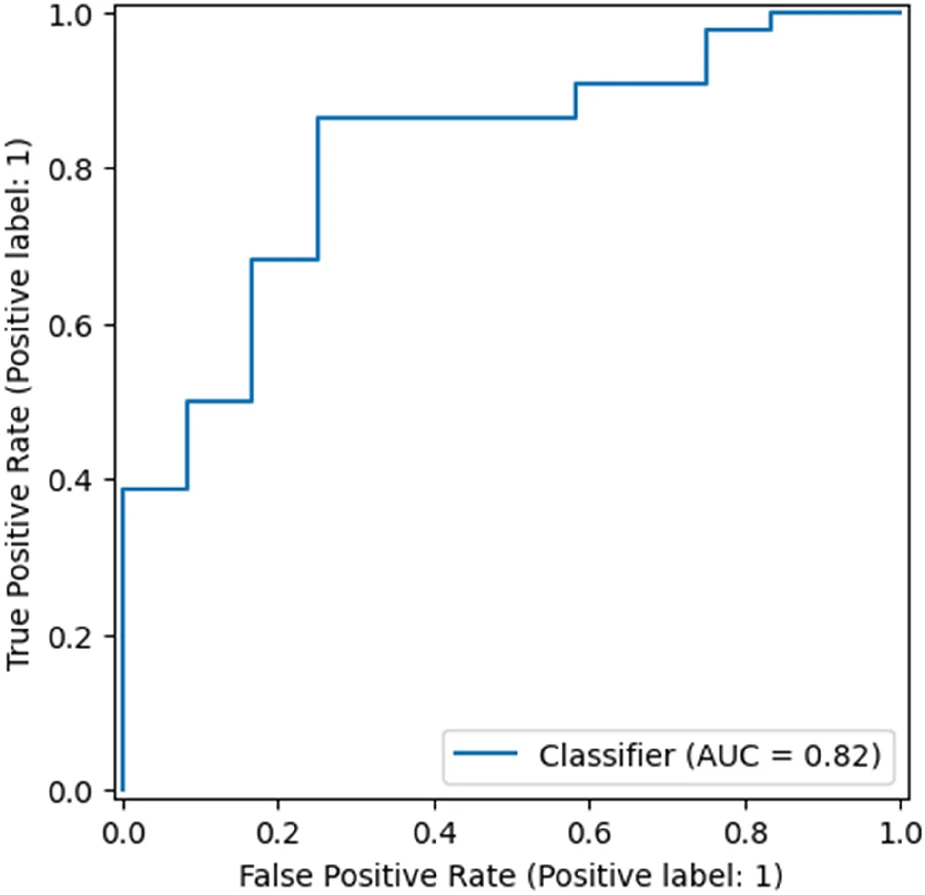
ROC curve and the achieved AUC result for RF based on the most important features.

Table 3 shows a comparison between the baseline RF model (using all input features) and enhanced RF model (using the selected features). The shift to the optimized feature set led to a meaningful boost in specificity, increasing from 33.33% to 66.67%, as well as a 21.54% enhancement in the F1-score for the “Not Improved” class (increasing from 40.00% to 61.54%). Even though the Area Under the Curve (AUC) revealed a slight decline (0.83 compared to 0.82), the enhanced model exhibited better average recall (Averaged recall) (76.52% as opposed to 62.12%) and an increased weighted average F1-score (82.62% vs. 76.89%). These findings suggest that eliminating low-impact variables allowed the model to attain a stronger discriminative ability between responders and non-responders. In clinical practice, this increased specificity signifies a significant change in approach. Instead of concentrating only on sensitivity (identifying those who will react), the improved specificity effectively reduces the chance of incorrect biologic placement by precisely identifying true non-responders prior to initiating treatment. Through the implementation of an objective screening layer, the decision support system (DSS) shields patients from a typical, expensive 4-to-6-month empirical trial-and-error phase with a biologic likely to be ineffective. Early identification of these non-responders speeds up the clinical shift to alternative, highly effective biological pathways, thus avoiding extended exposure to unmanaged symptoms and unwarranted drug side effects.

**Table 3:** Comparative summary between RF considering all input features and RF with selected features.

Metric	Using All Input Features	Using Selected Features
Accuracy	78.57%	**82** **.** **14%**
Sensitivity	**90**.**91%**	86.36%
Specificity	33.33%	**66**.**67%**
Average recall (Average of Sensitivity and Specificity)	62.12%	**76**.**52%**
Weighted average recall (Weighted Average of Sensitivity and Specificity)	78.57%	**82**.**14%**
Positive Predictive Value	83.33%	**90**.**48%**
Negative Predictive Value	50.00%	**57**.**14%**
Average Predictive Value	66.67%	**73**.**81%**
Weighted Average Predictive Value	76.19%	**83**.**33%**
F1-score (Improved)	86.96%	**88**.**37%**
F1-score (Not Improved)	40.00%	**61**.**54%**
Average F1-score	63.48%	**74**.**96%**
Weighted Average F1-score	76.89%	**82**.**62%**
AUC	**0**.**83**	0.82

Bold values indicate the better-performing result for each evaluation metric when comparing the Random Forest model using all input features with the Random Forest model using selected features.

As a result, the optimized RF model was chosen as the foundation for the Task 4 clinical recommender system because of its improved reliability and balanced predictive performance.

### 3.4 Prediction of optimal biological agent results (task 4)

The final task evaluated the RF model's capability to suggest the optimal biological agent by performing counterfactual simulation. This analysis acts as a “best effort” forecast, as the biological agent variable was shuffled for each patient in the test group while keeping all other clinical and demographic characteristics constant. This enabled the model to calculate the expected predicted probability of clinical improvement across four treatment categories: Omalizumab, Dupilumab, Mepolizumab, Benralizumab, and Tezepelumab.

As shown in the Supplementary Table S1, the model often indicated a strong probability of success for all agents, implying a wide therapeutic advantage for specific phenotypes. Nonetheless, notable differences in response probabilities that are clinically significant were noted in various cases. In a specific instance, a patient who started treatment with Omalizumab was designated as “Not Improved” by both the clinician and the model. The counterfactual simulation, however, suggested a 62% chance of improvement if the patient had started treatment with Benralizumab.

Although these findings offer a convincing proof-of-concept for AI-driven precision medicine, they still retain a predictive aspect. Absent prospective switching trials or long-term follow-up, these alternative results cannot be conclusively verified. However, these results highlight the model's ability to offer data-driven insights on optimal agent selection, which could lessen the “trial-and-error” efforts in managing severe asthma.

## 4 Discussion

To our knowledge, this is the first multi-country AI-driven decision support system informed by actual severe asthma biologic results (*n* = 801) adept at forecasting non-responders with significant accuracy and modeling hypothetical biologic switching situations. In contrast to typical machine learning models that often rely on specific, uniform clinical trial data, this framework explicitly incorporates diverse registry data from various continents. This distinctive data structure enables the system to stay highly predictive even with differences in regional demographics and localized healthcare systems.

This study demonstrates the transformative potential of AI-based decision support systems (DSS) in guiding personalized treatment for severe asthma patients. Using real-world clinical data from 801 patients with severe asthma from various healthcare systems. Utilizing enhanced Random Forest ensembles along with Recursive Feature Elimination, our model pinpointed ACT scores, baseline blood eosinophil levels, and particular Type-2 (T2) comorbidities mainly nasal polyposis and sinusitis as the key predictors of response to biological treatment. Incorporating this enhanced feature set directly resulted in a significant boost in performance, elevating the model's specificity to 66.67% while preserving high sensitivity and attaining an overall accuracy of 82.14%.These results align with earlier studies emphasizing the strength and precision of decision-tree based classification methods in clinical prediction tasks, particularly in managing imbalanced datasets and identifying nonlinear relationships among features (15–17).

The important features identified in the second task using RFE identified the baseline of FEV1, ACT, the number of exacerbations and oral corticosteroid (OCS) usage prior to administering biologics as a primary predictor of treatment success. Notably, although comorbidities such as GERD and OSA are conventionally associated with inadequate asthma management (18, 19), our model recognized them as minor features for forecasting biological efficacy. This indicates that although these conditions aggravate overall symptoms, they might not interfere with the specific molecular pathways (IL-5, IL-4/13) that biological therapies focus on. On the other hand, the significant predictive importance of sinusitis and nasal polyposis supports the “one-airway” theory, in which Type-2 inflammation in the upper airway acts as a strong indicator of biological responsiveness (20).

A key milestone in this research was the shift from the baseline model to the optimized feature set (Task 3). By applying RFE for feature selection optimizing the model by removing variables that had low less distinguishing power. This enhancement resulted in an improved overall accuracy of 82.14%, in contrast to the baseline model's 78.57%.

The most significant clinical enhancement was seen in the model's diagnostic specificity, which increased by double from 33.33% to 66.67%. Though the initial model showed strong sensitivity (90.91%), its poor specificity revealed a significant false-positive rate, indicating it often misidentified non-responders as “Improved”. Through the switch to the refined feature set, the model attained a more even predictive profile, greatly improving its dependability in detecting non-responders. Our system successfully bridges the persistent “endotype gap” in severe asthma treatment by converting complex, multi-faceted biological information into clear, actionable clinical insights. In everyday practice, healthcare professionals find it challenging to choose an accurate biologic when a patient shows overlapping T2-high traits (like increased IgE and eosinophils at the same time) or intricate T2-low profiles. Instead of depending on separate biomarker cutoffs, this AI system combines the complete clinical history and comorbidity profile simultaneously, transforming severe asthma management from a reactive, trial-and-error approach to a proactive, precision-medicine strategy. This differentiation holds clinical importance, as recognizing patients who are unlikely to gain from existing biologics can guide timely modifications in treatment approaches (21).

In the final task, we optimized the best performing model, namely RF model with feature selection, to perform counterfactual simulations, replicating different biological scenarios for each patient. Although based on statistical forecasts, this method reflects the tenets of precision medicine by anticipating possible reactions to various biological agents before initiation. Our evaluation suggests that switching agents could provide clinical advantages in specific patients, particularly when the model shows a significant contrast in predicated response rates. For instance, a patient with a suboptimal response to Omalizumab exhibited a 62% predicated probability for improvement if started on Benralizumab. This indicates that the model can recognize “non-responder phenotypes” that might be more appropriate for different inflammatory pathways (e.g., IL-5/5R vs. IgE). The counterfactual simulations presented in this study are predictive rather than causal. In routine clinical practice, biologic therapies are selected by clinicians based on patient characteristics such as biomarkers, disease severity, comorbidities, and prior treatment history. As a result, treatment assignment in the observational data is not random, and counterfactual “what-if” predictions may be influenced by confounding by indication, whereby the original biologic choice already reflects clinical judgment using similar information. Consequently, these simulations should not be interpreted as definitive estimates of treatment effect, but rather as hypothesis-generating decision-support outputs that require confirmation through prospective validation or causal-inference approaches (e.g., propensity-based adjustment or target-trial emulation). These results are consistent with recent studies promoting biomarker-based to enhance results in severe asthma which assisted in biological choice for severe asthma (22, 23). While AI-assisted decision-making in fields such as adult and pediatric oncology treatment (24); its application in severe asthma biologics treatment to our knowledge is not sufficiently explored. Nonetheless, the predictive method stays theoretical without direct clinical data and switching trials, highlighting the necessity for future validation (25).

A key insight from this study is the notable improvement of the model's clinical usefulness via feature optimization, suggests improved identification of non-responders; however, given the small independent test set, this finding should be considered preliminary and requires confirmation in larger external cohorts. Although accuracy typically dominates AI research, in biological therapy, identifying non-responders is essential to prevent months of costly and ineffective treatment. Nonetheless, the reality that 33% of non-responders are hard to categorize indicates that certain “refractory” phenotypes might be influenced by non-T2 pathways or environmental aspects that clinical data alone is unable to currently reflect.

This study has limitations despite its performance, such as its retrospective nature and the typical missing data points found in real-world medical registries. Incorporating cohorts from the UAE, Saudi Arabia, Poland, and Canada greatly improves generalizability, yet prospective validation in entirely independent geographical areas is necessary to verify overall adaptability. Upcoming research will aim to integrate this machine learning framework directly into real-time electronic health record (EHR) systems to assess its potential effects on physician choices and long-term patient health results. In summary, it should be recognized, nonetheless, that these probabilistic predictions are still theoretical. In the absence of direct longitudinal data from prospective switching trials, these findings act as a strong proof-of-concept instead of conclusive clinical evidence. However, this forecasting method underscores the ability of AI to close the existing gap in real-world evidence and advance beyond the conventional “trial-and-error” clinical model. However, no formal country- or site-stratified performance analyses were conducted, and the influence of dominant site-specific data distributions cannot be excluded.

### 4.1 Limitations and future work

#### 4.1.1 Limitations

This study has several limitations. First, the analysis is retrospective and relies on routinely collected registry data, which may include variable data completeness and unmeasured confounders. Second, although the cohort is multinational, it was assembled from a finite number of contributing centers/registries; therefore, the demographic and geographic representation may not fully reflect the broader severe-asthma population, and some degree of selection bias is possible. Third, the class imbalance (fewer non-responders) and the definition of “overall improvement” based on composite clinical criteria may influence model performance estimates and limit direct comparability with other studies using alternative endpoints. Because treatment allocation was determined by clinician judgment rather than randomization, counterfactual predictions may be affected by confounding by indication, further underscoring the need for prospective or causal-inference validation. Additionally, although the cohort is multinational, site- or country-specific validation was not performed, limiting assessment of model stability across healthcare systems.

Finally, the counterfactual switching results are model-based projections and cannot substitute for prospective switching trials or long-term outcome data. Formal paired statistical comparisons between Task 1 and Task 3 predictions (e.g., McNemar's test), as well as confidence intervals for performance metrics, were not conducted and should be addressed in future validation studies. In addition, although five-fold cross-validation was used during model development, final performance was evaluated using a single independent train/test split; therefore, repeated resampling with alternative splits or validation in fully external cohorts is required to confirm robustness and rule out split-specific effects.

#### 4.1.2 Future work

Future work will focus on prospective and external validation of the proposed decision support system in fully independent cohorts, including additional centers across different healthcare systems and ethnic backgrounds. Methodologically, evaluating alternative model architectures (e.g., gradient boosting variants, calibrated probabilistic models, and explainable frameworks) and incorporating longitudinal features (e.g., exacerbation trajectories and biomarker trends) may further improve discrimination of non-responder phenotypes. From an implementation perspective, integrating the tool into clinical workflows -such as embedding within electronic health record (EHR) environments with real-time data capture- will be essential to assess usability, clinician adoption, and impact on treatment decisions, time-to-response, and patient-relevant outcomes. Ultimately, pragmatic prospective studies and biologic switching trials informed by model recommendations are needed to confirm clinical benefit and safety. Finally, future work will focus on prospective and external validation of the proposed decision support system in fully independent cohorts, including repeated train/test splits and leave-one-site-out validation strategies.

## 5 Conclusion

Our study shows that Random Forest ensembles can successfully direct personalized treatment in severe asthma by forecasting clinical response with an average recall, reflecting both sensitivity and specificity, of 76.52%. Using an optimized set of features, we achieved a rise in specificity (from 33.33% to 66.67%), markedly improving the model's capacity to recognize non-responders and avert the extended use of inefficient, expensive biological therapies. Additionally, the model offered a data-driven approach for agent selection through counterfactual simulations, forecasting a 62% likelihood of improvement for certain patients by changing biologic classes. Although these probabilistic findings need future validation through prospective studies, they offer a strong proof-of-concept for moving from a “trial-and-error” clinical approach to AI-driven precision medicine in managing severe asthma.

## Code availability

The code used to generate the findings of this study is available from the corresponding author upon reasonable request.

## Data Availability

The datasets presented in this article are not readily available because the data for this study are from the International Severe Asthma Registry (ISAR) and are not available publicly. De-identified data may be available on reasonable request and approval by the relevant registry governance and participating institutions. Requests to access the datasets should be directed to lisalameh@yahoo.com.

## Supplementary material

Supplementary Table S1 Predicted response probabilities for alternative biologic agents generated using the optimized Random Forest model.
