# Supplementary material for: Utilizing AI for decision support in severe asthma biological treatment
Source: Front Digit Health. 2026 Oct 30;8:1939100. doi: 10.3389/fdgth.2026.1939100 (PMC13759770; doi:10.3389/fdgth.2026.1939100)
Supplement: Supplementary file 1 [file Table1.docx]

**Supplementary table 1:** Predicted response probabilities for alternative biologic agents generated using the optimized Random Forest model.

| **Biological Agent Currently Used** | **Current Status*** | **Predicted Status*** | **Probability of Improved Status** | **Predicted Status using Tezepelumab** | **Probability of Improved Status using Tezepelumab** | **Predicted Status using Omalizumab** | **Probability of Improved Status using Omalizumab** | **Predicted Status using Dupilumab** | **Probability of Improved Status using Dupilumab** | **Predicted Status using Mepolizumab** | **Probability of Improved Status using Mepolizumab** | **Predicted Status using Benralizumab** | **Probability of Improved Status using Benralizumab** |
| --- | --- | --- | --- | --- | --- | --- | --- | --- | --- | --- | --- | --- | --- |
| Benralizumab | 1 | 1 | 0.84 | 1 | 0.81 | 1 | 0.63 | 1 | 0.8 | 1 | 0.81 | 1 | 0.84 |
| Benralizumab | 1 | 1 | 0.79 | 1 | 0.79 | 1 | 0.61 | 1 | 0.78 | 1 | 0.78 | 1 | 0.79 |
| Mepolizumab | 0 | 1 | 0.56 | 1 | 0.57 | 1 | 0.55 | 1 | 0.6 | 1 | 0.56 | 1 | 0.58 |
| Benralizumab | 1 | 1 | 0.75 | 1 | 0.75 | 1 | 0.75 | 1 | 0.75 | 1 | 0.73 | 1 | 0.75 |
| Tezepelumab | 1 | 1 | 0.56 | 1 | 0.56 | 1 | 0.55 | 1 | 0.59 | 1 | 0.54 | 1 | 0.58 |
| Benralizumab | 1 | 1 | 0.54 | 1 | 0.53 | 1 | 0.51 | 1 | 0.57 | 1 | 0.52 | 1 | 0.54 |
| Benralizumab | 1 | 1 | 0.52 | 1 | 0.54 | 1 | 0.51 | 1 | 0.54 | 1 | 0.54 | 1 | 0.52 |
| Benralizumab | 1 | 1 | 0.65 | 1 | 0.64 | 1 | 0.62 | 1 | 0.67 | 1 | 0.64 | 1 | 0.65 |
| Mepolizumab | 0 | 1 | 0.61 | 1 | 0.64 | 1 | 0.64 | 1 | 0.64 | 1 | 0.61 | 1 | 0.65 |
| Dupilumab | 0 | 0 | 0.49 | 1 | 0.52 | 0 | 0.3 | 0 | 0.49 | 1 | 0.54 | 1 | 0.52 |
| Omalizumab | 1 | 1 | 0.8 | 1 | 0.81 | 1 | 0.8 | 1 | 0.8 | 1 | 0.78 | 1 | 0.82 |
| Benralizumab | 1 | 1 | 0.83 | 1 | 0.82 | 1 | 0.8 | 1 | 0.8 | 1 | 0.81 | 1 | 0.83 |
| Mepolizumab | 1 | 1 | 0.55 | 1 | 0.57 | 1 | 0.56 | 1 | 0.61 | 1 | 0.55 | 1 | 0.59 |
| Dupilumab | 1 | 1 | 0.56 | 1 | 0.56 | 1 | 0.54 | 1 | 0.56 | 1 | 0.54 | 1 | 0.56 |
| Benralizumab | 0 | 1 | 0.66 | 1 | 0.64 | 1 | 0.55 | 1 | 0.63 | 1 | 0.66 | 1 | 0.66 |
| Mepolizumab | 0 | 0 | 0.42 | 0 | 0.45 | 0 | 0.44 | 0 | 0.48 | 0 | 0.42 | 0 | 0.44 |
| Mepolizumab | 1 | 1 | 0.62 | 1 | 0.63 | 1 | 0.62 | 1 | 0.65 | 1 | 0.62 | 1 | 0.64 |
| Dupilumab | 1 | 0 | 0.34 | 0 | 0.2 | 0 | 0.18 | 0 | 0.34 | 0 | 0.18 | 0 | 0.19 |
| Dupilumab | 1 | 0 | 0.48 | 0 | 0.43 | 0 | 0.41 | 0 | 0.48 | 0 | 0.42 | 0 | 0.43 |
| Omalizumab | 1 | 1 | 0.61 | 1 | 0.62 | 1 | 0.61 | 1 | 0.62 | 1 | 0.61 | 1 | 0.63 |
| Mepolizumab | 0 | 0 | 0.13 | 0 | 0.16 | 0 | 0.14 | 0 | 0.33 | 0 | 0.13 | 0 | 0.16 |
| Benralizumab | 1 | 1 | 0.72 | 1 | 0.71 | 1 | 0.68 | 1 | 0.72 | 1 | 0.7 | 1 | 0.72 |
| Benralizumab | 1 | 1 | 0.61 | 1 | 0.61 | 1 | 0.6 | 1 | 0.61 | 1 | 0.59 | 1 | 0.61 |
| Mepolizumab | 1 | 1 | 0.55 | 1 | 0.56 | 1 | 0.55 | 1 | 0.59 | 1 | 0.55 | 1 | 0.57 |
| Mepolizumab | 1 | 1 | 0.54 | 1 | 0.57 | 1 | 0.55 | 1 | 0.59 | 1 | 0.54 | 1 | 0.6 |
| Benralizumab | 1 | 1 | 0.61 | 1 | 0.6 | 1 | 0.59 | 1 | 0.6 | 1 | 0.58 | 1 | 0.61 |
| Dupilumab | 1 | 1 | 0.7 | 1 | 0.7 | 1 | 0.67 | 1 | 0.7 | 1 | 0.67 | 1 | 0.7 |
| Mepolizumab | 1 | 1 | 0.79 | 1 | 0.8 | 1 | 0.79 | 1 | 0.8 | 1 | 0.79 | 1 | 0.8 |
| Omalizumab | 0 | 0 | 0.49 | 1 | 0.7 | 0 | 0.49 | 1 | 0.7 | 1 | 0.71 | 1 | 0.69 |
| Dupilumab | 1 | 0 | 0.35 | 0 | 0.25 | 0 | 0.23 | 0 | 0.35 | 0 | 0.21 | 0 | 0.26 |
| Benralizumab | 1 | 1 | 0.92 | 1 | 0.91 | 1 | 0.85 | 1 | 0.9 | 1 | 0.9 | 1 | 0.92 |
| Omalizumab | 1 | 1 | 0.63 | 1 | 0.7 | 1 | 0.63 | 1 | 0.71 | 1 | 0.7 | 1 | 0.71 |
| Omalizumab | 1 | 1 | 0.85 | 1 | 0.85 | 1 | 0.85 | 1 | 0.85 | 1 | 0.83 | 1 | 0.85 |
| Omalizumab | 1 | 1 | 0.52 | 1 | 0.66 | 1 | 0.52 | 1 | 0.65 | 1 | 0.65 | 1 | 0.66 |
| Omalizumab | 1 | 1 | 0.61 | 1 | 0.67 | 1 | 0.61 | 1 | 0.67 | 1 | 0.65 | 1 | 0.67 |
| Dupilumab | 0 | 0 | 0.49 | 0 | 0.45 | 0 | 0.39 | 0 | 0.49 | 0 | 0.45 | 0 | 0.45 |
| Mepolizumab | 1 | 0 | 0.29 | 0 | 0.32 | 0 | 0.29 | 0 | 0.38 | 0 | 0.29 | 0 | 0.33 |
| Benralizumab | 1 | 1 | 0.68 | 1 | 0.68 | 1 | 0.68 | 1 | 0.68 | 1 | 0.66 | 1 | 0.68 |
| Dupilumab | 1 | 0 | 0.48 | 0 | 0.46 | 0 | 0.42 | 0 | 0.48 | 0 | 0.45 | 0 | 0.46 |
| Dupilumab | 1 | 0 | 0.34 | 0 | 0.25 | 0 | 0.23 | 0 | 0.34 | 0 | 0.21 | 0 | 0.26 |
| Omalizumab | 0 | 0 | 0.28 | 1 | 0.51 | 0 | 0.28 | 1 | 0.51 | 1 | 0.55 | 1 | 0.52 |
| Mepolizumab | 0 | 0 | 0.31 | 0 | 0.35 | 0 | 0.3 | 0 | 0.42 | 0 | 0.31 | 0 | 0.35 |
| Benralizumab | 1 | 1 | 0.76 | 1 | 0.76 | 1 | 0.62 | 1 | 0.75 | 1 | 0.76 | 1 | 0.76 |
| Dupilumab | 1 | 1 | 0.73 | 1 | 0.73 | 1 | 0.73 | 1 | 0.73 | 1 | 0.71 | 1 | 0.72 |
| Omalizumab | 0 | 0 | 0.42 | 1 | 0.6 | 0 | 0.42 | 1 | 0.59 | 1 | 0.61 | 1 | 0.62 |
| Omalizumab | 0 | 1 | 0.51 | 1 | 0.54 | 1 | 0.51 | 1 | 0.56 | 1 | 0.53 | 1 | 0.54 |
| Dupilumab | 1 | 1 | 0.57 | 1 | 0.56 | 1 | 0.55 | 1 | 0.57 | 1 | 0.55 | 1 | 0.56 |
| Omalizumab | 1 | 1 | 0.76 | 1 | 0.77 | 1 | 0.76 | 1 | 0.76 | 1 | 0.76 | 1 | 0.77 |
| Dupilumab | 1 | 1 | 0.57 | 1 | 0.56 | 1 | 0.55 | 1 | 0.57 | 1 | 0.54 | 1 | 0.57 |
| Mepolizumab | 1 | 1 | 0.58 | 1 | 0.6 | 1 | 0.6 | 1 | 0.6 | 1 | 0.58 | 1 | 0.61 |
| Tezepelumab | 1 | 1 | 0.58 | 1 | 0.58 | 1 | 0.52 | 1 | 0.58 | 1 | 0.58 | 1 | 0.58 |
| Benralizumab | 1 | 1 | 0.82 | 1 | 0.82 | 1 | 0.82 | 1 | 0.81 | 1 | 0.81 | 1 | 0.82 |
| Omalizumab | 1 | 1 | 0.68 | 1 | 0.72 | 1 | 0.68 | 1 | 0.72 | 1 | 0.7 | 1 | 0.73 |
| Benralizumab | 1 | 1 | 0.6 | 1 | 0.58 | 1 | 0.55 | 1 | 0.62 | 1 | 0.57 | 1 | 0.6 |
| Omalizumab | 1 | 1 | 0.63 | 1 | 0.67 | 1 | 0.63 | 1 | 0.68 | 1 | 0.65 | 1 | 0.68 |
| Mepolizumab | 1 | 1 | 0.83 | 1 | 0.83 | 1 | 0.77 | 1 | 0.81 | 1 | 0.83 | 1 | 0.83 |
